# Postbiotics as Promising Tools for Cancer Adjuvant Therapy

**DOI:** 10.34172/apb.2021.007

**Published:** 2020-11-07

**Authors:** Aziz Homayouni Rad, Leili Aghebati Maleki, Hossein Samadi Kafil, Hamideh Fathi Zavoshti, Amin Abbasi

**Affiliations:** ^1^Nutrition Research Center, Tabriz University of Medical Sciences, Tabriz, Iran.; ^2^Immunology Research Center, Tabriz University of Medical Sciences, Tabriz, Iran.; ^3^Drug Applied Research Center, Tabriz University of Medical Sciences, Tabriz, Iran.; ^4^Department of Food Hygiene and Aquatics, Faculty of Veterinary Medicine, Tabriz University, Tabriz, Iran.; ^5^Student’s Research Committee, Tabriz University of Medical Sciences, Tabriz, Iran.

**Keywords:** Cancer, Postbiotic, Probiotic, Health, Treatment

## Abstract

As many investigations have reported, there is a complicated relation between fermented foods, lactic acid bacteria (LAB), and human health. It seems that bioactive components such as prebiotics, probiotics, and postbiotics are key mediators of the complex and direct association between these factors. LAB activity in the matrix of fermented foods and improving their growth by prebiotic compounds ultimately results in the production of bioactive molecules (postbiotics), which possess specific biological and physiological properties. The term "postbiotics" refers to a complex of biological micro- and macromolecules, if consumed in adequate amounts, provides the host with different health-promoting effects. Different reports have suggested that postbiotics possess the ability to moderate the effectiveness of cancer treatment and reduce the side-effects of conventional therapies in cancer patients due to their anti-proliferative, anti-inflammatory and anti-cancer properties. Consequently, postbiotics, for their unique characteristics, have gained great scientific attention and are considered as a novel approach for adjuvant therapy in patients with cancer.

Lactic acid bacteria (LAB) are an integral part of foods and have been present in the matrix of fermented foods as safe and functional compounds whose consumption is directly linked to health.^1^ LAB in the matrix of fermented foods, utilizes appropriate substrates (prebiotics) and subsequently, generates a wide range of bioactive substances such as organic acids and bacteriocins, which possess key biological and physiological properties.^2^

The main pro-health effects of LAB and its derived bioactive metabolites include (a) improving gastrointestinal disorders such as inflammatory bowel diseases (IBD), (b) improving urogenital disorders, (c) improving lactose metabolism, (d) antidiabetic activities, (e) immunomodulation properties, (f) anticarcinogenic activities, (g) improving lactose metabolism, and (h) promoting cancer therapies.^3-5^ In this regard, a body of epidemiological pieces of evidence confirms the positive effects of LAB and its derived metabolites on the colon, bladder, liver, breast, and gastric cancers.^6^

The anticarcinogenic effect of LAB is mediated through different mechanisms including gut microbiota modification and dominance of beneficial microbiota, improvement of the immune system function/response, and possessing significant antioxidative and anti-proliferative properties.^7^ Moreover, other clinical health merits of LAB and its metabolites associated with the cancer therapies include the establishment of eubiosis conditions in the gut ecosystem, contribution to the recovery (after cancer surgery) and decreasing hospitalization period,^8^ eliminating superficial incisional surgical position infection,^9^ and preventing some side-effects of conventional cancer therapies (chemotherapy and antibiotic-induced diarrhea).^10,11^ Several factors such as virulence factor and antibiotic resistance genes transfer to host’s (humans, animals) pathogenic microbes,^12^ stimulating acute inflammatory responses^13^ and significant difference between the declared levels with the actual amount of live probiotic cells in commercial products^14^ have made great interests in probiotic health effects mediated by beneficial microbial metabolites which are characterized as postbiotics.

The term of postbiotics refers to a complex of micro- and macromolecules such as inactivated microbial cells (non-viable cells), cell fractions (muropeptides, teichoic acids, endo- and exopolysaccharides, and surface-layer proteins) or cell metabolites (short-chain fatty acids, SCFAs), organic acids, bacteriocins, and enzymes) that are naturally made by live probiotic cells in fermentation process and/or made synthetically by laboratory procedures. If they are consumed in sufficient quantities, they can leave different physiological health-promoting effects on the consumer.^15,16^ On the other hand, postbiotics are known as multi-functional agents owning anti-microbial, anti-inflammatory, anti-oxidant, immunomodulation, anti-hypertensive, anti-diabetic, anti-obesogenic, and anti-proliferative activities, which are attributed to the presence of surface and intracellular bioactive molecules.^17-20^ In this regard, Gao et al^21^ evaluated the biological role of gut beneficial microbiota-derived postbiotics in preserving gut health and function. They established that postbiotics can act as their parent live cells and can be considered as a safe alternative to live probiotic cells.

The application of postbiotics as an adjunct for cancer prevention and treatment was strongly associated with the function/response of the host immune system. Different investigations confirmed the potential role of postbiotics in the prevention and treatment of cancer, particularly in gastrointestinal (GI) cancer cases^22,23^ (Table 1). In this regard, Motevaseli et al^33^ reported the selectively anti-proliferative effects of various postbiotics (whole inactivated cell, cell-wall, and cytoplasmic extracts, culture supernatants) derived from vaginal-origin *Lactobacillus crispatus* and *L. gasseri* on normal and cervical tumor cells. The most highlighted feature of postbiotics is their ability to distinguish between normal and cancer cells, which modulates the proliferation of normal cells but suppresses angiogenesis and drives apoptosis in cancerous cells.^34^ Ou et al^35^ investigated the effect of diet on colon cancer risk. They studied the gut microbiota through their metabolites in humans with high (African Americans) and low risk (rural native Africans) of colon cancer. They found notable associations between reduced generations of SCFAs, increased secondary bile acid metabolites, and increased risk of colon cancer. Their findings confirmed that colon cancer risk can be affected by the balance between the microbial generation of potentially health-promoting and carcinogenic metabolites. Besides, the postbiotic of exopolysaccharide (EPS) derived from *Lactobacillus* spp is reported to exert significant anti-proliferative activities against colonic carcinoma cell lines.^36^ The cell-free supernatants (CFS) derived from human breast milk *L. casei* and *L. paracasei* have anti-carcinogenic effects against cervical cancer cell lines.^37^ Shyu et al.^38^ evaluated the cytotoxic effects of *Lactobacillus* spp-derived CFS (isolated from dairy products) on colon cancer cells (HCT116 and HT-29), leukemia cells (THP-1), and normal human dermal fibroblasts (HDFn) with PrestoBlue. They established that all studied probiotic CFSs have cytotoxic effects on HCT-116 and HT-29 colon cancer cell lines. They also considerably up-regulated the expression of early apoptotic-promoting *cfos*, *cjun* and down-regulated the proinflammatory cytokine *IL-β*, *TNF-* α genes in treated cancer cells with CFSs. The outcomes clearly supported the potential application of postbiotics in the modulation of inflammatory responses (as a precursor to carcinogenesis) and anticancer therapy. Further mechanisms involved in the anti-cancer activities of postbiotics include regulating immune responses, reducing cell viability, binding to mutagenic and carcinogenic constituents, triggering pro-apoptotic cell death pathways, reducing microbial translocation, increasing apoptosis and necrosis, increasing tumor cell death via autophagy, anti-proliferative activity against cancer cells, decreasing metalloproteinase-9 activity, and inhibiting cancer invasion.^39-42^ Therefore, it is clear that gut beneficial microbes-derived postbiotic components have significant anti-proliferative properties due to their potentiality in regulating cell cycle, stimulating differentiation, and up-regulating the pro-apoptotic pathways in different cancer cells. These biological properties are mainly based on the phenotypic mood of cells, the parent microbial cell strains, methods applied to the preparation of postbiotics, and the presence of bioactive micro- and macromolecules^43^ (Figure 1).

**Table 1:** Postbiotics and their potential anti-cancer activities

**Bacteria**	**Inactivation method**	**Postbiotic**	**Cell line(s)**	**Main mechanism(s)**	**Reference**
*Lactobacillus rhamnosus* SHA111, SHA112, and SHA113	S^*^	CFS	HeLa	Induction of apoptosis by up-regulation of BAD, BAX, Caspase-3, Caspase-8, Caspase-9, and down-regulation of BCL-2 genes	^24^
*Lactobacillus fermentum* sp	S	CFS	HCT-116, HT-29	Induction of apoptosis by up-regulation of Caspase-3, Bax, Bak, Noxa, and Bid mRNA expressions	^25^
*Lactobacillus casei* ATCC334	S	CFS, Ferrichrome	SW-620	Induction of apoptosis by the activation of c-jun N-terminal kinase	^26^
*Bifidobacterium* spp	S	CFS	SW-742	Decrease cell proliferation	^27^
*Lactobacillus plantarum* GD2, *Lactobacillus rhamnosus* E9, *Lactobacillus brevis* LB63, and *Lactobacillus delbrueckii* ssp. *bulgaricus* B3	TT^**^	EPS	HT-29	Induction of apoptosis via increasing the expression of Bax, Caspase-3, Caspase-9 and decreasing the expression of Bcl-2 and Survivin	^28^
*Faecalibacterium prausnitzii* A2–165	S	CFS, EV	A549	Up-regulate anti-inflammatory cytokines (IL-10, TGF-β2 and IL-1Ra) and down-regulate some of the important pro-inflammatory cytokines such as IL-6, TNF-𝛼 and TNF-β	^29^
*Lactobacillus paracasei* sp	S	CWP	Caco-2	Decrease cell proliferation Induction of apoptosis	^30^
*Lactobacillus plantarum* sp	S	CFS	MCF-7	Induction of apoptosis via increasing the expression of DECAY, FADD, RAS64B apoptotic genes and decreasing the expression of BCL-2 and BUFFY genes	^31^
*Clostridium butyricum* sp	S	SCFA	HCT-116, Caco-2, and HCT-8	Suppresses the Wnt/β-catenin signaling pathway and modulate the gut microbiota composition	^32^

CFS: cell-free supernatant, EPS: exopolysaccharide, EV: extracellular vesicles, CWP: cell wall protein, SCFA: short-chain fatty acid. Cervical cancer cells: HeLa. Colon cancer cells: HT-29, Caco-2, SW-620, SW-742, HCT-8, HCT-116. Breast cancer cells: MCF-7. Lung adenocarcinoma epithelial cells: A549. *Sonication, **Thermal treatment.

**Figure 1:**
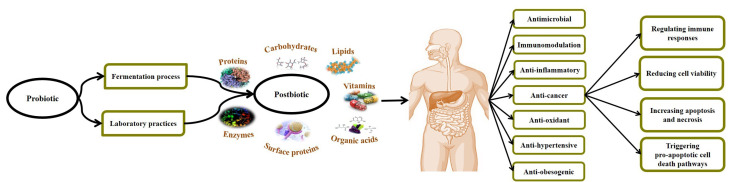
Postbiotics and their potential anti-cancer effects in the host.

In conclusion, due to their unique characteristics (safe origin, more shelf-life, low preparation cost, without toxic effect), postbiotics have received much scientific attention and are considered as a novel approach for adjuvant therapy in patients with cancer. Randomized double-blind clinical trials are necessary to specify the optimal dose and administration frequency of postbiotic supplements for cancer patients.

## Ethical Issues

Not applicable

## Conflicts of Interest

The authors declare that they have no conflicts of interest.

## Acknowledgments

The authors gratefully acknowledge the financial support of this study by the Tabriz University of Medical Sciences, Tabriz, Iran.
